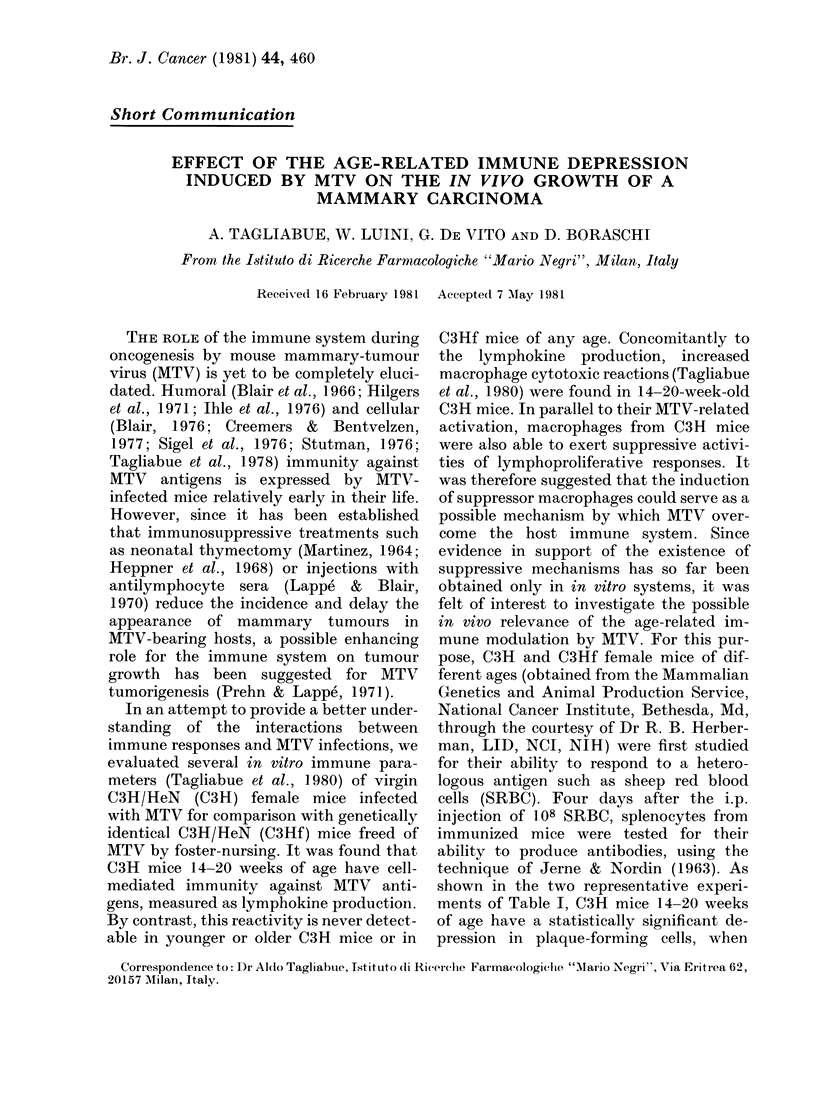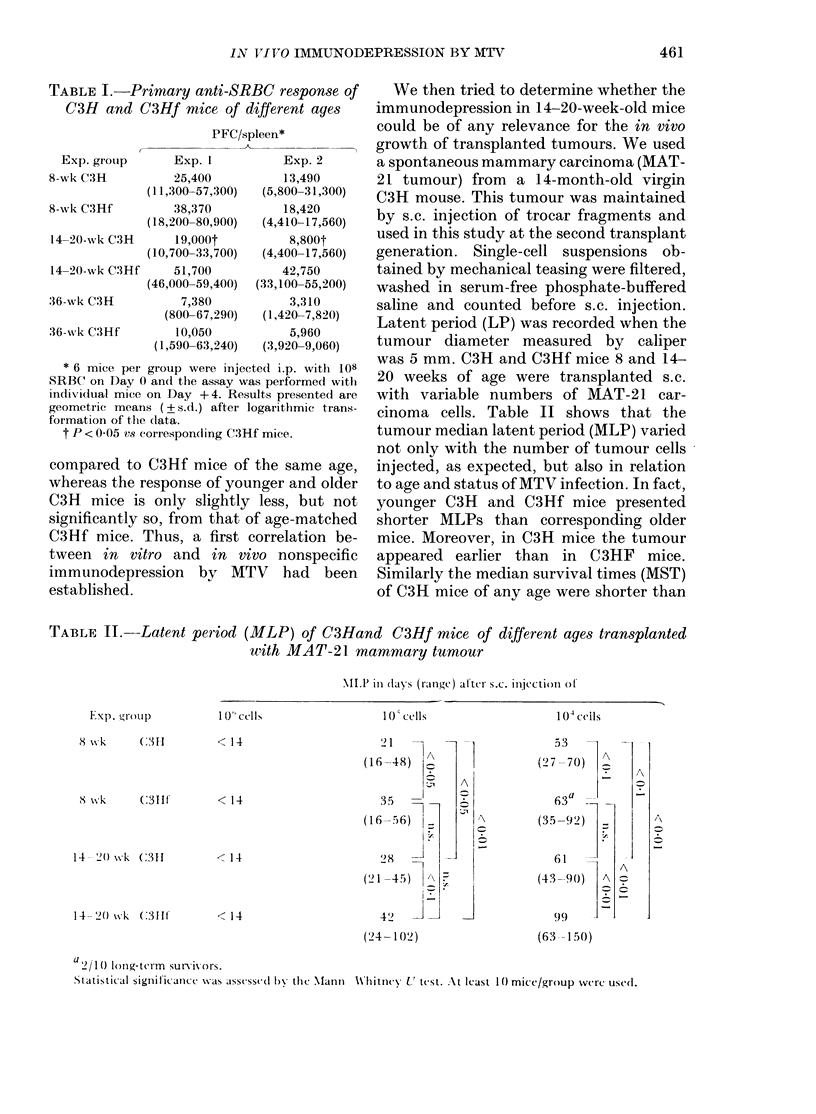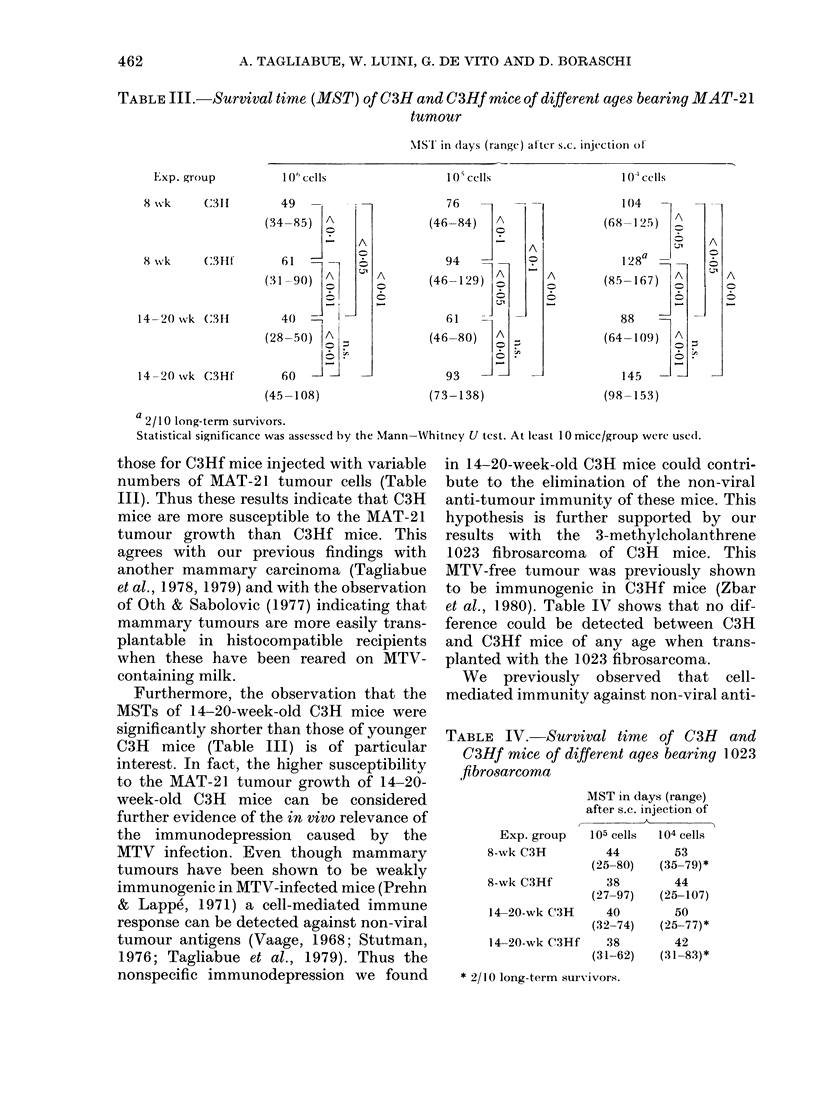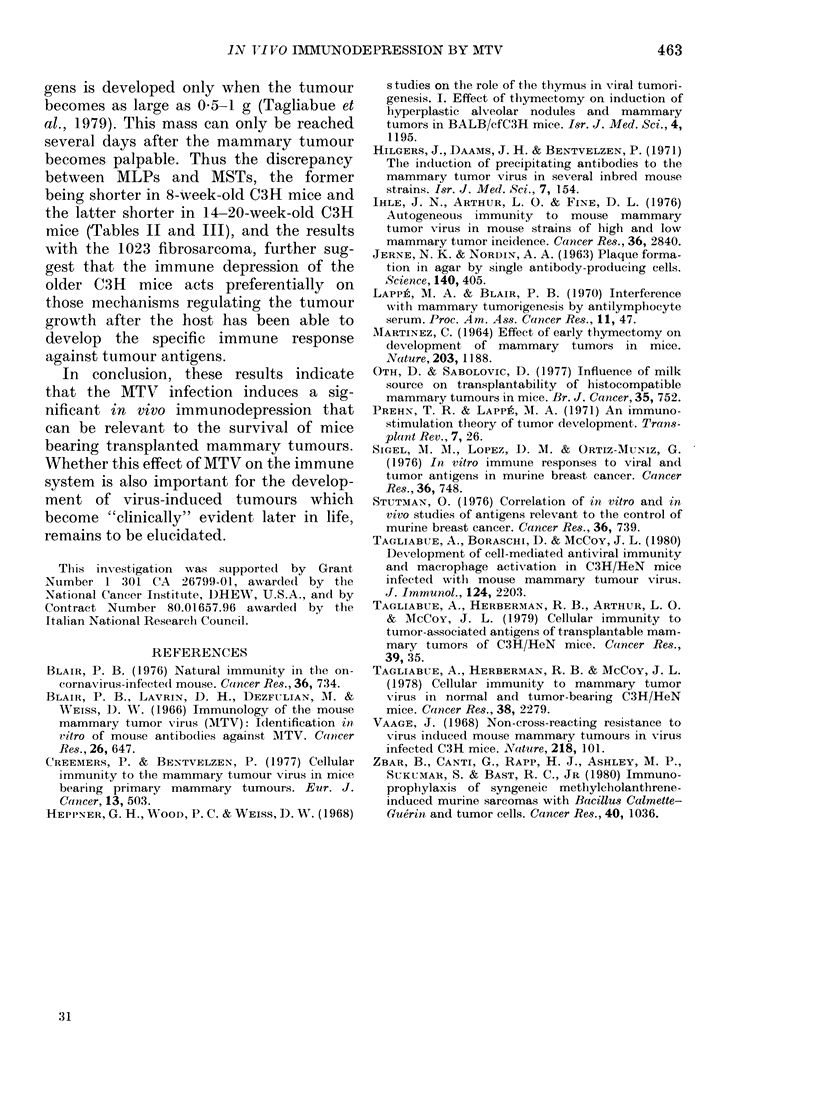# Effect of the age-related immune depression induced by MTV on the in vivo growth of a mammary carcinoma.

**DOI:** 10.1038/bjc.1981.206

**Published:** 1981-09

**Authors:** A. Tagliabue, W. Luini, G. De Vito, D. Boraschi


*Br. J. Cancer* (1981) **44,** 460

***Short Communication***

### EFFECT OF THE AGE-RELATED IMMUNE DEPRESSION INDUCED BY MTV ON THE *IN VIVO* GROWTH OF A MAMMARY CARCINOMA

A. TAGLIABUE, W. LUINI, G. DE VITO AND D. BORASCHI

*From the Istituto di Ricerche Farmacologiche "Mario Negri", Milan, Italy*

Received 16 February 1981 Accepted 7 May 1981

THE ROLE of the immune system during oncogenesis by mouse mammary-tumour virus (MTV) is yet to be completely elucidated. Humoral (Blair *et al.*, 1966; Hilgers *et al.*, 1971; Ihle *et al.*, 1976) and cellular (Blair, 1976; Creemers & Bentvelzen, 1977; Sigel *et al.*, 1976; Stutman, 1976; Tagliabue *et al.*, 1978) immunity against MTV antigens is expressed by MTV-infected mice relatively early in their life. However, since it has been established that immunosuppressive treatments such as neonatal thymectomy (Martinez, 1964; Heppner *et al.*, 1968) or injections with antilymphocyte sera (Lappé & Blair, 1970) reduce the incidence and delay the appearance of mammary tumours in MTV-bearing hosts, a possible enhancing role for the immune system on tumour growth has been suggested for MTV tumorigenesis (Prehn & Lappé, 1971).

In an attempt to provide a better understanding of the interactions between immune responses and MTV infections, we evaluated several *in vitro* immune parameters (Tagliabue *et al.*, 1980) of virgin C3H/HeN (C3H) female mice infected with MTV for comparison with genetically identical C3H/HeN (C3Hf) mice freed of MTV by foster-nursing. It was found that C3H mice 14–20 weeks of age have cell-mediated immunity against MTV antigens, measured as lymphokine production. By contrast, this reactivity is never detectable in younger or older C3H mice or in C3Hf mice of any age. Concomitantly to the lymphokine production, increased macrophage cytotoxic reactions (Tagliabue *et al.*, 1980) were found in 14–20-week-old C3H mice. In parallel to their MTV-related activation, macrophages from C3H mice were also able to exert suppressive activities of lymphoproliferative responses. It was therefore suggested that the induction of suppressor macrophages could serve as a possible mechanism by which MTV overcome the host immune system. Since evidence in support of the existence of suppressive mechanisms has so far been obtained only in *in vitro* systems, it was felt of interest to investigate the possible *in vivo* relevance of the age-related immune modulation by MTV. For this purpose, C3H and C3Hf female mice of different ages (obtained from the Mammalian Genetics and Animal Production Service, National Cancer Institute, Bethesda, Md, through the courtesy of Dr R. B. Herberman, LID, NCI, NIH) were first studied for their ability to respond to a heterologous antigen such as sheep red blood cells (SRBC). Four days after the i.p. injection of $10^8$ SRBC, splenocytes from immunized mice were tested for their ability to produce antibodies, using the technique of Jerne & Nordin (1963). As shown in the two representative experiments of Table I, C3H mice 14–20 weeks of age have a statistically significant depression in plaque-forming cells, when

Correspondence to: Dr Aldo Tagliabue, Istituto di Ricerche Farmacologiche "Mario Negri", Via Eritrea 62, 20157 Milan, Italy.

TABLE I.—*Primary anti-SRBC response of C3H and C3Hf mice of different ages*

| Exp. group | PFC/spleen* Exp. 1 | Exp. 2 |
|---|---|---|
| 8-wk C3H | 25,400 (11,300–57,300) | 13,490 (5,800–31,300) |
| 8-wk C3Hf | 38,370 (18,200–80,900) | 18,420 (4,410–17,560) |
| 14–20-wk C3H | 19,000† (10,700–33,700) | 8,800† (4,400–17,560) |
| 14–20-wk C3Hf | 51,700 (46,000–59,400) | 42,750 (33,100–55,200) |
| 36-wk C3H | 7,380 (800–67,290) | 3,310 (1,420–7,820) |
| 36-wk C3Hf | 10,050 (1,590–63,240) | 5,960 (3,920–9,060) |

* 6 mice per group were injected i.p. with $10^8$ SRBC on Day 0 and the assay was performed with individual mice on Day +4. Results presented are geometric means (±s.d.) after logarithmic transformation of the data.

† $P < 0{\cdot}05$ *vs* corresponding C3Hf mice.

compared to C3Hf mice of the same age, whereas the response of younger and older C3H mice is only slightly less, but not significantly so, from that of age-matched C3Hf mice. Thus, a first correlation between *in vitro* and *in vivo* nonspecific immunodepression by MTV had been established.

We then tried to determine whether the immunodepression in 14–20-week-old mice could be of any relevance for the *in vivo* growth of transplanted tumours. We used a spontaneous mammary carcinoma (MAT-21 tumour) from a 14-month-old virgin C3H mouse. This tumour was maintained by s.c. injection of trocar fragments and used in this study at the second transplant generation. Single-cell suspensions obtained by mechanical teasing were filtered, washed in serum-free phosphate-buffered saline and counted before s.c. injection. Latent period (LP) was recorded when the tumour diameter measured by caliper was 5 mm. C3H and C3Hf mice 8 and 14–20 weeks of age were transplanted s.c. with variable numbers of MAT-21 carcinoma cells. Table II shows that the tumour median latent period (MLP) varied not only with the number of tumour cells injected, as expected, but also in relation to age and status of MTV infection. In fact, younger C3H and C3Hf mice presented shorter MLPs than corresponding older mice. Moreover, in C3H mice the tumour appeared earlier than in C3HF mice. Similarly the median survival times (MST) of C3H mice of any age were shorter than

TABLE II.—*Latent period (MLP) of C3Hand C3Hf mice of different ages transplanted with MAT-21 mammary tumour*

| Exp. group | | MLP in days (range) after s.c. injection of $10^6$ cells | $10^5$ cells | $10^4$ cells |
|---|---|---|---|---|
| 8 wk | C3H | < 14 | 21 (16–48) | 53 (27–70) |
| 8 wk | C3Hf | < 14 | 35 (16–56) | 63[a] (35–92) |
| 14–20 wk | C3H | < 14 | 28 (21–45) | 61 (43–90) |
| 14–20 wk | C3Hf | < 14 | 42 (24–102) | 99 (63–150) |

| Comparison | $10^5$ cells | $10^4$ cells |
|---|---|---|
| 8 wk C3H vs 8 wk C3Hf | <0·05 | <0·1 |
| 8 wk C3Hf vs 14–20 wk C3H | n.s. | n.s. |
| 14–20 wk C3H vs 14–20 wk C3Hf | <0·1 | <0·01 |
| 8 wk C3Hf vs 14–20 wk C3Hf | n.s. | <0·01 |
| 8 wk C3H vs 14–20 wk C3H | <0·05 | <0·1 |
| 8 wk C3H vs 14–20 wk C3Hf | <0·01 | <0·01 |

[a] 2/10 long-term survivors.

Statistical significance was assessed by the Mann Whitney *U* test. At least 10 mice/group were used.

TABLE III.—*Survival time (MST) of C3H and C3Hf mice of different ages bearing MAT-21 tumour*

| Exp. group | MST in days (range) after s.c. injection of $10^6$ cells | $10^5$ cells | $10^4$ cells |
|---|---|---|---|
| 8 wk C3H | 49 (34–85) | 76 (46–84) | 104 (68–125) |
| 8 wk C3Hf | 61 (31–90) | 94 (46–129) | 128[a] (85–167) |
| 14–20 wk C3H | 40 (28–50) | 61 (46–80) | 88 (64–109) |
| 14–20 wk C3Hf | 60 (45–108) | 93 (73–138) | 145 (98–153) |

| Comparison | $10^6$ cells | $10^5$ cells | $10^4$ cells |
|---|---|---|---|
| 8 wk C3H vs 8 wk C3Hf | $< 0·1$ | $< 0·1$ | $< 0·05$ |
| 8 wk C3Hf vs 14–20 wk C3H | $< 0·01$ | $< 0·05$ | $< 0·01$ |
| 14–20 wk C3H vs 14–20 wk C3Hf | $< 0·01$ | $< 0·01$ | $< 0·01$ |
| 8 wk C3H vs 14–20 wk C3H | $< 0·05$ | $< 0·1$ | $< 0·05$ |
| 8 wk C3Hf vs 14–20 wk C3Hf | n.s. | n.s. | n.s. |
| 8 wk C3H vs 14–20 wk C3Hf | $< 0·01$ | $< 0·01$ | $< 0·01$ |

[a] 2/10 long-term survivors.
Statistical significance was assessed by the Mann–Whitney *U* test. At least 10 mice/group were used.

those for C3Hf mice injected with variable numbers of MAT-21 tumour cells (Table III). Thus these results indicate that C3H mice are more susceptible to the MAT-21 tumour growth than C3Hf mice. This agrees with our previous findings with another mammary carcinoma (Tagliabue *et al.*, 1978, 1979) and with the observation of Oth & Sabolovic (1977) indicating that mammary tumours are more easily transplantable in histocompatible recipients when these have been reared on MTV-containing milk.

Furthermore, the observation that the MSTs of 14–20-week-old C3H mice were significantly shorter than those of younger C3H mice (Table III) is of particular interest. In fact, the higher susceptibility to the MAT-21 tumour growth of 14–20-week-old C3H mice can be considered further evidence of the *in vivo* relevance of the immunodepression caused by the MTV infection. Even though mammary tumours have been shown to be weakly immunogenic in MTV-infected mice (Prehn & Lappé, 1971) a cell-mediated immune response can be detected against non-viral tumour antigens (Vaage, 1968; Stutman, 1976; Tagliabue *et al.*, 1979). Thus the nonspecific immunodepression we found in 14–20-week-old C3H mice could contribute to the elimination of the non-viral anti-tumour immunity of these mice. This hypothesis is further supported by our results with the 3-methylcholanthrene 1023 fibrosarcoma of C3H mice. This MTV-free tumour was previously shown to be immunogenic in C3Hf mice (Zbar *et al.*, 1980). Table IV shows that no difference could be detected between C3H and C3Hf mice of any age when transplanted with the 1023 fibrosarcoma.

We previously observed that cell-mediated immunity against non-viral anti-

TABLE IV.—*Survival time of C3H and C3Hf mice of different ages bearing* 1023 *fibrosarcoma*

| Exp. group | MST in days (range) after s.c. injection of $10^5$ cells | $10^4$ cells |
|---|---|---|
| 8-wk C3H | 44 (25–80) | 53 (35–79)* |
| 8-wk C3Hf | 38 (27–97) | 44 (25–107) |
| 14–20-wk C3H | 40 (32–74) | 50 (25–77)* |
| 14–20-wk C3Hf | 38 (31–62) | 42 (31–83)* |

* 2/10 long-term survivors.

gens is developed only when the tumour becomes as large as 0·5–1 g (Tagliabue *et al.*, 1979). This mass can only be reached several days after the mammary tumour becomes palpable. Thus the discrepancy between MLPs and MSTs, the former being shorter in 8-week-old C3H mice and the latter shorter in 14–20-week-old C3H mice (Tables II and III), and the results with the 1023 fibrosarcoma, further suggest that the immune depression of the older C3H mice acts preferentially on those mechanisms regulating the tumour growth after the host has been able to develop the specific immune response against tumour antigens.

In conclusion, these results indicate that the MTV infection induces a significant *in vivo* immunodepression that can be relevant to the survival of mice bearing transplanted mammary tumours. Whether this effect of MTV on the immune system is also important for the development of virus-induced tumours which become "clinically" evident later in life, remains to be elucidated.

This investigation was supported by Grant Number 1 301 CA 26799-01, awarded by the National Cancer Institute, DHEW, U.S.A., and by Contract Number 80.01657.96 awarded by the Italian National Research Council.

### REFERENCES

BLAIR, P. B. (1976) Natural immunity in the oncornavirus-infected mouse. *Cancer Res.*, **36**, 734.

BLAIR, P. B., LAVRIN, D. H., DEZFULIAN, M. & WEISS, D. W. (1966) Immunology of the mouse mammary tumor virus (MTV): Identification *in vitro* of mouse antibodies against MTV. *Cancer Res.*, **26**, 647.

CREEMERS, P. & BENTVELZEN, P. (1977) Cellular immunity to the mammary tumour virus in mice bearing primary mammary tumours. *Eur. J. Cancer*, **13**, 503.

HEPPNER, G. H., WOOD, P. C. & WEISS, D. W. (1968) Studies on the role of the thymus in viral tumorigenesis. I. Effect of thymectomy on induction of hyperplastic alveolar nodules and mammary tumors in BALB/cfC3H mice. *Isr. J. Med. Sci.*, **4**, 1195.

HILGERS, J., DAAMS, J. H. & BENTVELZEN, P. (1971) The induction of precipitating antibodies to the mammary tumor virus in several inbred mouse strains. *Isr. J. Med. Sci.*, **7**, 154.

IHLE, J. N., ARTHUR, L. O. & FINE, D. L. (1976) Autogeneous immunity to mouse mammary tumor virus in mouse strains of high and low mammary tumor incidence. *Cancer Res.*, **36**, 2840.

JERNE, N. K. & NORDIN, A. A. (1963) Plaque formation in agar by single antibody-producing cells. *Science*, **140**, 405.

LAPPÉ, M. A. & BLAIR, P. B. (1970) Interference with mammary tumorigenesis by antilymphocyte serum. *Proc. Am. Ass. Cancer Res.*, **11**, 47.

MARTINEZ, C. (1964) Effect of early thymectomy on development of mammary tumors in mice. *Nature*, **203**, 1188.

OTH, D. & SABOLOVIC, D. (1977) Influence of milk source on transplantability of histocompatible mammary tumours in mice. *Br. J. Cancer*, **35**, 752.

PREHN, T. R. & LAPPÉ, M. A. (1971) An immunostimulation theory of tumor development. *Transplant Rev.*, **7**, 26.

SIGEL, M. M., LOPEZ, D. M. & ORTIZ-MUNIZ, G. (1976) *In vitro* immune responses to viral and tumor antigens in murine breast cancer. *Cancer Res.*, **36**, 748.

STUTMAN, O. (1976) Correlation of *in vitro* and *in vivo* studies of antigens relevant to the control of murine breast cancer. *Cancer Res.*, **36**, 739.

TAGLIABUE, A., BORASCHI, D. & MCCOY, J. L. (1980) Development of cell-mediated antiviral immunity and macrophage activation in C3H/HeN mice infected with mouse mammary tumour virus. *J. Immunol.*, **124**, 2203.

TAGLIABUE, A., HERBERMAN, R. B., ARTHUR, L. O. & MCCOY, J. L. (1979) Cellular immunity to tumor-associated antigens of transplantable mammary tumors of C3H/HeN mice. *Cancer Res.*, **39**, 35.

TAGLIABUE, A., HERBERMAN, R. B. & MCCOY, J. L. (1978) Cellular immunity to mammary tumor virus in normal and tumor-bearing C3H/HeN mice. *Cancer Res.*, **38**, 2279.

VAAGE, J. (1968) Non-cross-reacting resistance to virus induced mouse mammary tumours in virus infected C3H mice. *Nature*, **218**, 101.

ZBAR, B., CANTI, G., RAPP, H. J., ASHLEY, M. P., SUKUMAR, S. & BAST, R. C., JR (1980) Immunoprophylaxis of syngeneic methylcholanthrene-induced murine sarcomas with *Bacillus Calmette–Guérin* and tumor cells. *Cancer Res.*, **40**, 1036.